# Immunotherapy-Based Combinations in First-Line Urothelial Cancer: A Systematic Review and Individual Patient Data (IPD) Meta-Analysis

**DOI:** 10.3390/curroncol31080352

**Published:** 2024-08-20

**Authors:** Mattia Alberto Di Civita, Andrea Torchia, Daniele Santini, Daniele Marinelli, Virginia Magro, Marianna Cerro, Laura Pappalardo, Giulia Maltese, Fiorenza Santamaria, Luca Zacco, Dorelsa Buccilli, Ailin Dehghanpour, Iolanda Speranza, Alessandro Sciarra, Valeria Panebianco, Michela Roberto

**Affiliations:** 1 Department of Experimental Medicine, Sapienza University, 00161 Rome, Italy; mattiaalberto.dicivita@uniroma1.it (M.A.D.C.); daniele.marinelli@uniroma1.it (D.M.); fiorenza.santamaria@uniroma1.it (F.S.); 2 Department of Radiological Sciences, Oncology and Pathology, Sapienza University of Rome, 00185 Rome, Italy; laura.pappalardo@uniroma1.it (L.P.); giulia.maltese@uniroma1.it (G.M.); luca.zacco@uniroma1.it (L.Z.); dorelsa.buccilli@uniroma1.it (D.B.); ailin.dehghanpour@uniroma1.it (A.D.); valeria.panebianco@uniroma1.it (V.P.); 3 Clinical and Molecular Medicine, Sapienza University of Rome, 00185 Rome, Italy; andrea.torchia@uniroma1.it (A.T.); marianna.cerro@uniroma1.it (M.C.); 4 Division of Medical Oncology A, Policlinico Umberto I, 00161 Rome, Italy; iolandasperanza@libero.it (I.S.); mikiroberto87@gmail.com (M.R.); 5 Department of Medical-Surgical Sciences and Biotechnologies, Sapienza University, 04100 Latina, Italy; 6 Faculty of Medicine and Surgery, Sapienza University, 00161 Rome, Italy; magro.1740894@studenti.uniroma1.it; 7 Division of Medical Oncology B, Policlinico Umberto I, 00161 Rome, Italy; 8 Department of Urology, Sapienza Rome University, Policlinico Umberto I, 00161 Rome, Italy; alessandro.sciarra@uniroma1.it

**Keywords:** antibody–drug conjugate, enfortumab vedotin, nivolumab, pembrolizumab, atezolizumab

## Abstract

Introduction. Platinum-based chemotherapy represents the standard of care (SoC) for the first-line treatment of advanced urothelial carcinoma (mUC). The benefit of adding immune checkpoint inhibitors (ICIs) to platinum-based chemotherapy was recently investigated. We performed an individual patient data (IPD) meta-analysis of phase 3 clinical trials comparing ICI-based treatments. Methods. A systematic literature search was conducted on the MEDLINE and CENTRAL databases. The results were filtered by including only reports on clinical trials or randomized clinical trials from 2018 to 2023, including 3047 patients from four clinical trials (EV302, CHECKMATE-901, IMVIGOR130, KEYNOTE-361). An IPD meta-analysis was performed by reconstructing IPD from Kaplan–Meier curves. The primary endpoints were overall survival (OS) and progression-free survival (PFS) of Pembrolizumab + EV compared to experimental arms of the other trials of immunotherapy + chemotherapy. Results. The OS analysis showed an advantage of IPD from EV302 vs. all the other trials. For EV302 vs. KEYNOTE-361, the HR was 0.51; for EV302 vs. IMVIGOR130, the HR was 0.47; and for EV302 vs. CHECKMATE-901, the HR was 0.66 (CI 95% 0.51–0.85). In the PFS analysis, the EV302 arm showed a statistically significant advantage compared to CHECKMATE-901 (HR 0.66) and versus IMVIGOR130 (HR 0.51). Limitations: By using reconstructed IPD curves, it was not possible to adjust patient-level covariates, and the heterogeneity of the included population may have affected the pooled results. Conclusions: The EV302 experimental arm showed better OS and PFS when compared to the other immunochemotherapy combinations. An immunochemotherapy combination strategy at the beginning of treatment in mUC seems to be superior in terms of OS and PFS compared to platinum-based chemotherapy alone. EV–Pembrolizumab resulted to have better outcomes compared to avelumab, rather than other immunochemotherapy combinations. However, given the heterogeneity of these studies, a longer follow up and prospective trials are needed to confirm these data.

## 1. Introduction

Platinum-based chemotherapy represents the standard of care (SoC) for the first-line treatment of unresectable or metastatic urothelial carcinoma (mUC) in platinum-eligible patients [1]. Despite the effectiveness of platinum-based treatment regimens, long-lasting positive responses are infrequent, and nearly half of patients diagnosed with advanced urothelial carcinoma cannot receive cisplatin chemotherapy due to cisplatin ineligibility (an ECOG (Eastern Cooperative Oncology Group) performance status of 2 and/or creatinine clearance < 60 mL/min and/or Common Terminology Criteria for Adverse Events (CTCAE) Gr ≥ 2 hearing loss and/or CTCAE Gr ≥ 2 neuropathy); the remaining half of the patients are treated with carboplatin and about 10% of the patients are also carboplatin ineligible [2,3,4].

In recent decades, the treatment landscape for advanced urothelial carcinoma has undergone a significant transformation with the advent of immune checkpoint inhibitors (ICIs) and antibody–drug conjugates (ADCs).

In the first-line metastatic setting, the use of ICIs has been investigated in phase three trials, either in combination with chemotherapy or as maintenance therapy, against the SoC, in some cases offering improvements in the duration of response (DOR), progression-free survival (PFS), and overall survival (OS) [5,6,7,8,9,10].

Pembrolizumab and Atezolizumab received approval as a monotherapy from both the Food and Drug Administration (FDA) and the European Medicines Agency (EMA) as first-line treatments in patients ineligible for platinum-based chemotherapy with programmed death ligand 1 (PD-L1) expression, as well as patients who are not eligible for any platinum-based chemotherapy, regardless of PD-L1 status [11].

Recently, the phase III CheckMate (CM)-901 trial evaluated the combination of Nivolumab plus Gemcitabine–cisplatin versus Gemcitabine–cisplatin chemotherapy in patients with previously untreated unresectable or metastatic urothelial carcinoma, revealing significantly improved OS and PFS compared to the SoC, with a median DOR of 37.1 months with Nivolumab combination therapy and 13.2 months with only a chemotherapy regimen [9].

Despite the promising results of combination therapy involving ICIs, phase III trials examining the integration of chemotherapy with either Pembrolizumab or Durvalumab in the first-line setting failed to produce encouraging outcomes [10,12].

Enfortumab vedotin (EV), an ADC directed against nectin-4, demonstrated a substantial extension in survival compared to the SoC as a second- or third-line treatment for advanced urothelial carcinoma [13].

Preclinical findings and promising clinical results have led to its investigation in settings involving earlier stages of the disease in association with ICIs [14,15].

The combination of EV plus Pembrolizumab in cisplatin-ineligible patients has exhibited promising outcomes as a first-line therapy for metastatic urothelial carcinoma, as it demonstrated a manageable safety profile, a DOR of 25.6 months (mo), and a median OS of 26.1 mo in the phase Ib/II trial [16].

These findings have recently been validated in the phase III trial EV302/KEYNOTE-A39, where the EV–Pembrolizumab combination was assessed against chemotherapy with Gemcitabine and either cisplatin or carboplatin (based on their eligibility to receive cisplatin therapy) as a first-line treatment; the combination significantly improved outcomes, nearly doubling the median PFS and OS versus standard platinum-based chemotherapy [16].

Notwithstanding the availability of several treatment options and/or strategies (ICI with chemotherapy in combination or the sequential schedule), there is a lack of clarity concerning the optimal first-line treatment strategy for mUC. Aiming to address this gap, we performed a systematic review and individual patient data (IPD) meta-analysis of phase 3 clinical trials comparing ICI-based therapies (combined with platinum chemotherapy or with EV) with the SoC as first-line treatments in patients affected with mUC.

Moreover, we conducted an exploratory analysis to assess the survival curve of patients treated with Pembrolizumab + Enfortumab and other first-line immunotherapy-based treatment strategies with avelumab maintenance. 

## 2. Methods 

### 2.1. Inclusion Criteria

In this individual patient data (IPD) meta-analysis, we included phase 3 randomized controlled trials (RCTs) regarding first-line treatment for advanced urothelial cell carcinoma (including both upper and lower tract), reporting on the efficacy and safety of immune checkpoint inhibitor (ICI)-based treatment, in combination with chemotherapy. We only included clinical trials enrolling platinum-eligible patients; therefore, patients in the control arm of the included trials received either cisplatin or carboplatin in combination with Gemcitabine. Early-phase and non-randomized clinical trials were excluded from the present analysis. We included only studies published in the English language.

### 2.2. Search Strategy and Selection Processes

A systematic literature search was conducted on the MEDLINE and CENTRAL databases in December 2023 by MADC. We used Medical Subject Headings (MeSH) for the MEDLINE and CENTRAL database and selected keywords; the results were filtered by including only reports on clinical trials or randomized clinical trials from 2018 to 2023. For each outcome we included the most recent viable report.

The following variables were extracted from all included studies: first authors, year of first publication, study name (NCT number), eligibility criteria, treatment arms, number of patients enrolled in each arm, number of cisplatin-eligible patients, and study endpoints (Table 1).

PFS was defined as the time from randomization to imaging-based progression or death, whichever occurred first. OS was defined as the time from randomization to death.

Safety was assessed with any grade treatment-related adverse events. Adverse events (AEs) grading was performed with the CTCAE criteria.

## 3. Endpoints

### 3.1. Primary Endpoints

- OS and PFS of Pembrolizumab + EV compared to experimental arms of the other trials of immunotherapy + chemotherapy.

### 3.2. Secondary Endpoints

- Safety in selected studies.
- OS and PFS in patients enrolled in studies assessing the efficacy of immunochemotherapy combinations in first-line settings compared with chemotherapy arms.
- OS and PFS of the included experimental arms of clinical trials versus avelumab (data from JAVELIN BLADDER 101).

## 4. Data Extraction

We reconstructed the IPD from Kaplan–Meier curves using the IPDfromKM workflow (UR: https://biostatistics.mdanderson.org/shinyapps/IPDfromKM/ accessed on 5 January 2024). Three authors (MADC, DM, AT) independently performed the extraction. Summary statistics (median PFS, landmark analyses) were used to compare original and reconstructed data, and the most accurate extraction was selected for subsequent analyses.

### Statistical Analysis

Survival curves were estimated using the Kaplan–Meier product-limit method and were compared using the log-rank test. Hazard ratios (HRs) and relative 95% confidence intervals (95% CIs) were estimated with Cox regression analyses. Odds ratios (ORs) and 95% CIs were estimated with a Mantel–Haenszel random effects model.

This analysis was conducted in accordance with the PRISMA (Preferred Reporting Items for Systematic Reviews and Meta-Analyses) guidelines.

The analyses were performed with the software R, version 4.1, with the following packages: survival, survminer. The safety data analyses were performed with Revman Web version 5.3.1.

For the primary endpoint of comparing EV–Pembrolizumab versus the other immunochemotherapy regimens, we firstly compared the OS and PFS of the control-arm IPD curves to evaluate if the control arms had similar outcomes. Concerning OS (Figures S2 and S3), all standard of care arms had a comparable median OS, except for IMVIGOR131, whose standard of care arm performed worse than others (HR 1.24 CI 95 1.01–1.5 vs. SOC arm of EV302 taken as reference). For PFS, the standard of care arm of KEYNOTE (KN)-361 performed better than the others (Figures S4 and S5), with an HR of 0.48 (CI 95% 0.39–0.6 vs. EV302) taken as reference. Assuming a statistically similar prognosis for the standard of care arms of all the trials, we performed an analysis of the IPD curves, comparing the OS and PFS of Pembrolizumab plus EV compared singularly to the other experimental curves of chemotherapy plus immunotherapy.

For the secondary endpoint of safety analysis, we compared the incidence of adverse events (neutropenia, anemia, cutaneous rash, and pruritus) in the experimental arms of the included trials versus the control arm, by using the odds ratio.

For the endpoint of comparing OS and PFS data from the phase 3 immunochemotherapy combination versus standard of care arms, considering the homogeneity of patient selection from all the control arms in each trial (excluding CheckMate-901 which enrolled only cisplatin-eligible patients), we decided to create a control-arm KM curve which contained the IPD of the control arms (standard of care chemotherapy) merged together. We compared this KM curve with the ones obtained from the IPD of each experimental arm of the included trials for both PFS and OS.

For the exploratory endpoint of comparing data from the immunochemotherapy combination versus immunotherapy maintenance, we removed from the analysis all the patients progressing in the first 4 months to select the patients who did not progress after the first disease evaluation, making the included population as similar as possible to that of JAVELIN BLADDER 101 (in which they enrolled only patients who did not progress after the first 4–6 cycles of chemotherapy). After doing this, we compared the IPD KM curves of OS and PFS from the included trials’ experimental arms versus the avelumab IPD KM curves.

## 5. Results

### 5.1. Included Studies

**Figure 1:**
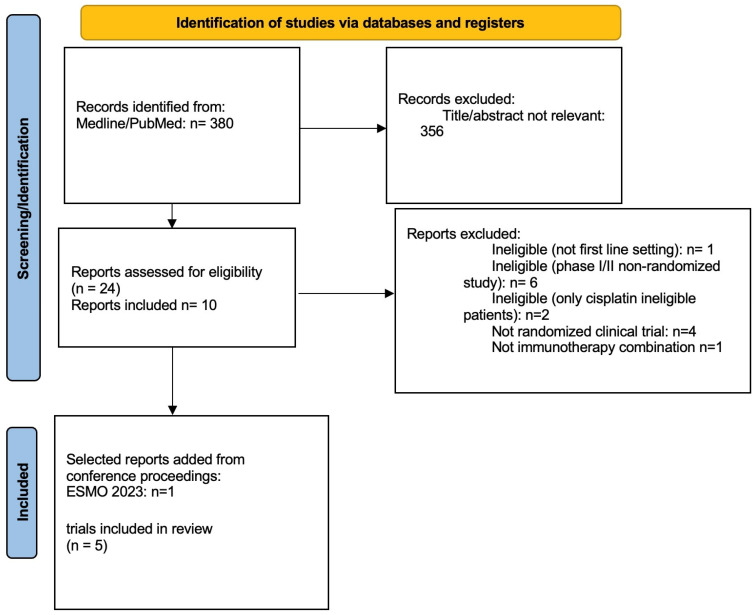
PRISMA flow diagram of the study.

The systematic literature search identified 380 records; after exclusion of duplicate and not relevant reports, 10 reports from 5 studies were included (EV302 (17), IMVIGOR130 [6,7], KEYNOTE-361 [13], CHECKMATE-901 [9], JAVELIN BLADDER 101 [8]) (Figure 1).

The total number of patients evaluated in this analysis is 3047. All the included studies were phase 3, randomized clinical trials. In all the included studies, immunotherapy was administered with platinum-based chemotherapy, except for EV302, where the experimental arm was Pembrolizumab + Enfortumab vedotin.

CHECKMATE-901 is the only trial in which only cisplatin-eligible patients were enrolled. In any case, the median OS and PFS of the standard of care arm are similar to the ones of the other studies (Figures S1–S4). The IMVIGOR130 and KEYNOTE-361 trials included three arms: immunotherapy–chemotherapy combination vs. immunotherapy vs. standard of care chemotherapy; for both trials, we analyzed only the immunotherapy plus chemotherapy arms, excluding from our analysis the mono immunotherapy arm.

For the exploratory endpoint in which we compared the individual patient data from the included clinical trials with the one of JAVELIN BLADDER 101, we included 1735 patients for OS and 1515 for PFS.

### 5.2. Pembrolizumab Plus Enfortumab Vedotin vs. Immunotherapy + Chemotherapy

**Figure 2:**
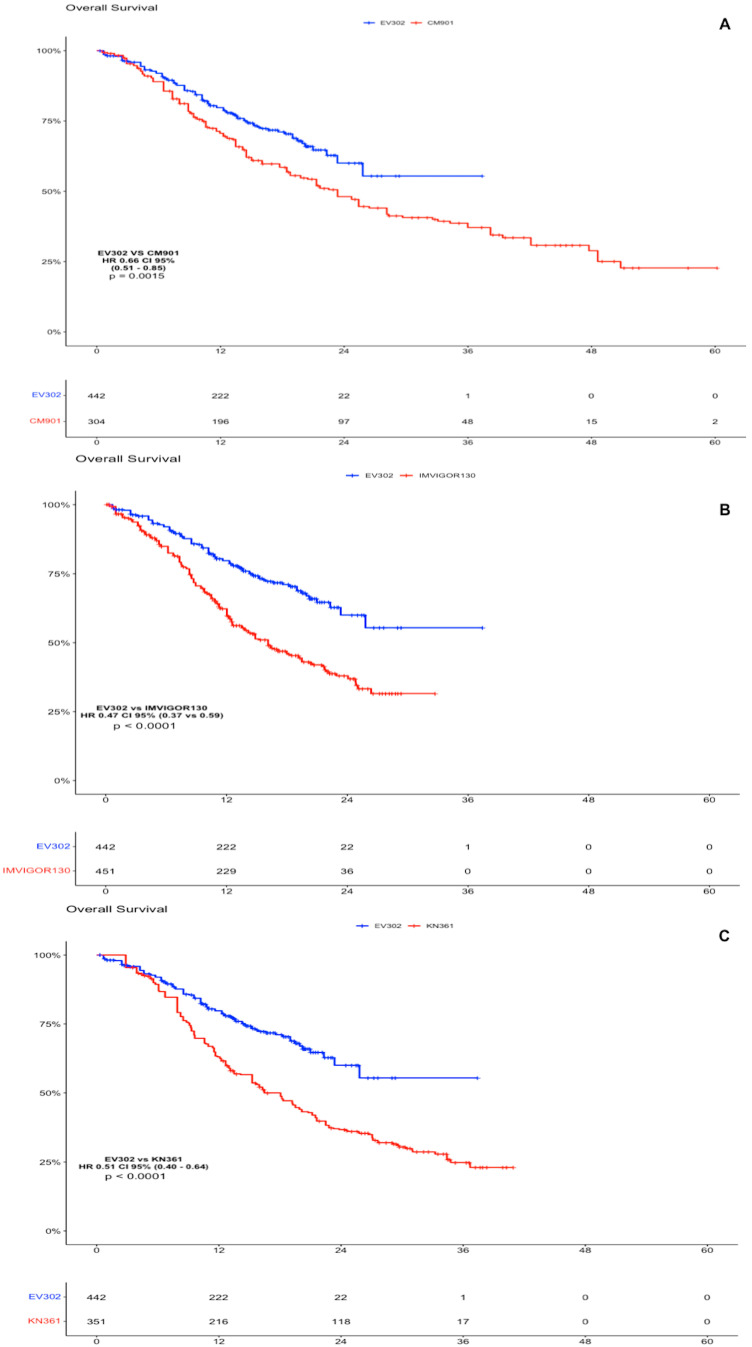
OS analysis of experimental arms of EV302 vs. chemotherapy plus immunotherapy trials. (**A**). Kaplan–Meier analysis of OS for EV302 vs. CM-901. (**B**). Kaplan–Meier analysis of OS for EV302 vs. IMVIGOR130. (**C**). Kaplan–Meier analysis of OS for EV302 vs. KN-361. OS: overall survival; PFS: progression-free survival; CM-901: CheckMate-901; KN-361: KEYNOTE-361.

Considering the absence of platinum-based chemotherapy in the EV arm, we decided to compare the individual patient data obtained from the Kaplan–Meier curve for the OS and PFS of EV302 vs. the other clinical trials. As far as OS goes, the analysis showed a statistically significant advantage of IPD from EV302 vs. all the other trials. For EV302 vs. KEYNOTE-361, the HR was 0.51 (CI 95% 0.40–0.64, *p* < 0.0001); for EV302 vs. IMVIGOR130, the HR was 0.47 (CI 95% 0.37–0.59, *p* < 0.0001); and for EV302 vs. CHECKMATE-901, the HR was 0.66 (CI 95% 0.51–0.85, *p* = 0.0015) (Figure 2).

**Figure 3:**
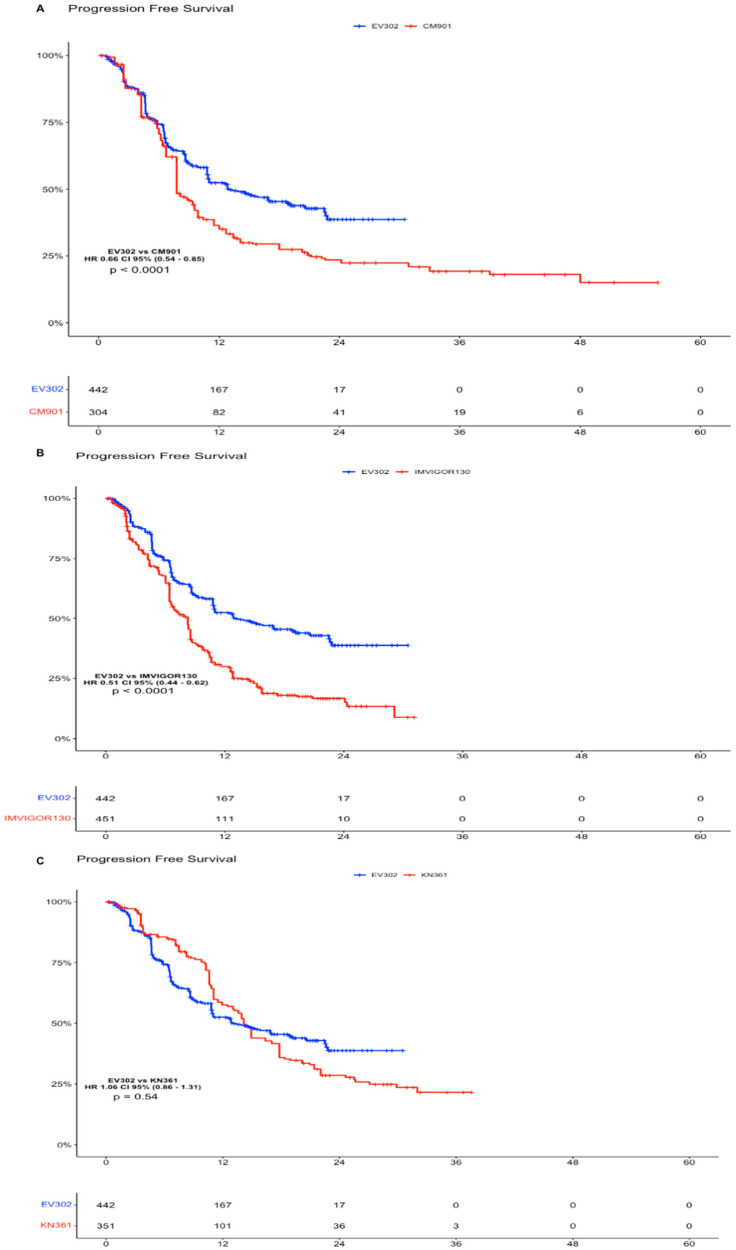
Progression-Free Survival analysis of experimental arms of EV302 vs. chemotherapy plus immunotherapy trials. (**A**). Kaplan–Meier analysis of PFS for EV302 vs. CM-901. (**B**). Kaplan–Meier analysis of PFS for EV302 vs. IMVIGOR130. (**C**). Kaplan–Meier analysis of OS for EV302 vs. KN-361. OS: overall survival; PFS: progression-free survival; CM-901: CheckMate-901; KN-361: KEYNOTE-361.

In the PFS analysis, the EV302 arm showed a statistically significant advantage compared to CHECKMATE-901 (HR 0.66 CI 95% 0.54–0.85, *p* < 0.0001) and versus IMVIGOR130 (HR 0.51, CI 95% 0.44–0.62, *p* < 0.0001). There was no significant difference in terms of PFS comparing individual patient data of EV302 versus KEYNOTE-361 (HR 1.06, CI 95% 0.86–1.31, *p* = 0.54), but we have to note that the standard of care arms of KEYNOTE-361 performed better than the other, leading us to think that there was a different selection of patients in this trial (Figure 3).

### 5.3. Secondary Endpoint

#### 5.3.1. Overall Survival

**Figure 4:**
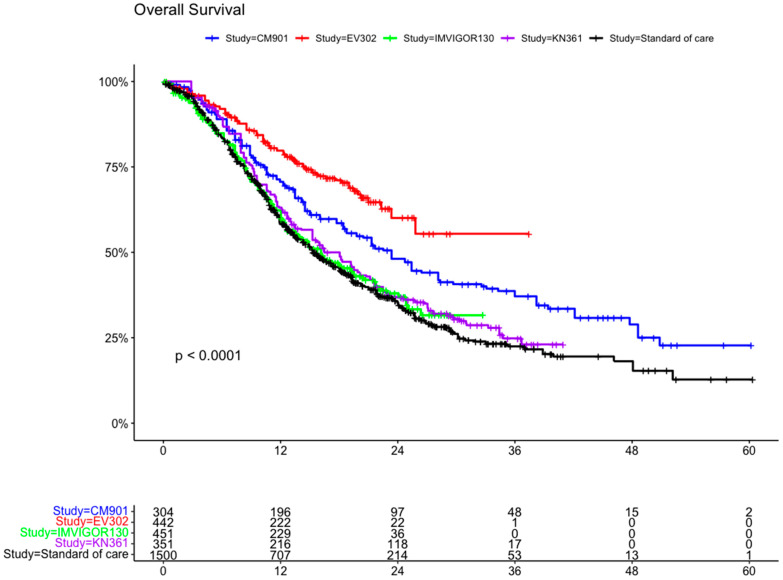
Kaplan–Meier curve for overall survival in the ITT population of the included trials. ITT: intention to treat; CM-901: CheckMate-901; KN-361: KEYNOTE-361.

**Figure 5:**
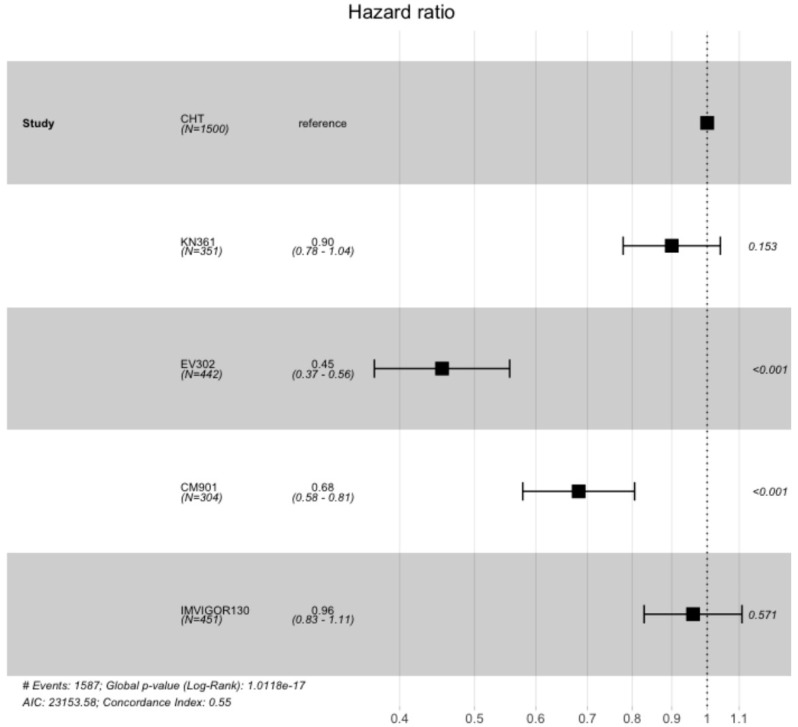
Hazard ratios comparing overall survival of the included trial comparing experimental arm vs. standard of care arm (which included individual patient data from all included trials). OS: overall survival; PFS: progression-free survival; CM-901: CheckMate-901; KN-361: KEYNOTE-361.

A total of 3047 patients from four clinical trials were included in the OS analysis (1547 patients in the experimental arms and 1500 in the control arms). In the intention to treat (ITT) population, the experimental arms showed an advantage compared with the SoC arm (which included the individual patient data for OS from all the control arms (platinum-based chemotherapy) of the clinical trials) (Figure 4 and Figure 5). The advantage was statistically significant for EV302 (HR 0.45, CI 95% 0.37–0.56, *p* < 0.001) and CHECKMATE-901 (HR 0.68 CI 0.58–0.81 *p* < 0.001) but not statistically significant for KEYNOTE-361 (HR 0.90 CI 95% 0.78–1.04 *p* = 0.153) and IMVIGOR130 (HR 0.96 CI 0.83–1.11 *p* = 0.571).

#### 5.3.2. Progression-Free Survival

**Figure 6:**
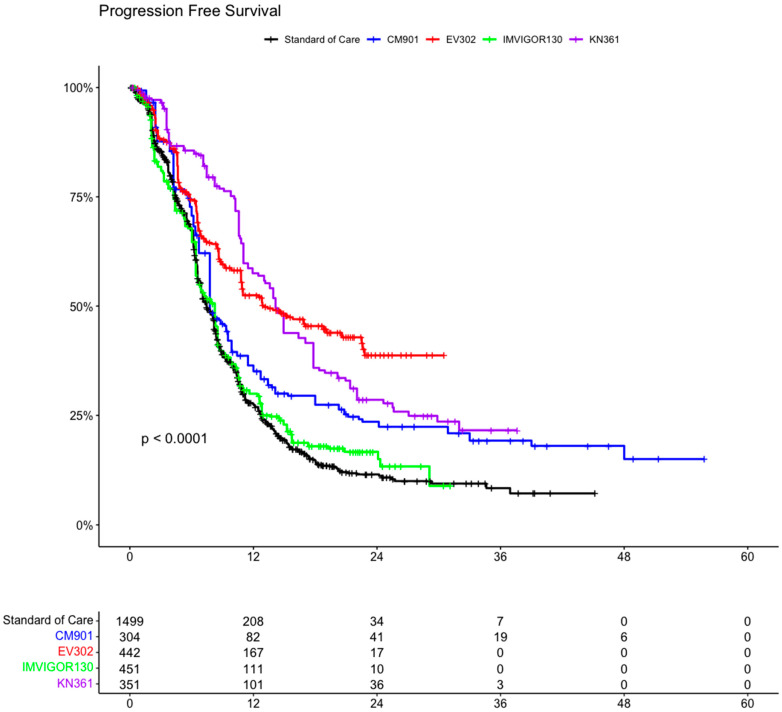
Kaplan–Meier curve of progression-free survival of ITT population in the included trials. ITT: intention to treat; CM-901: CheckMate-901; KN-361: KEYNOTE-361.

In the PFS analysis, we included 3047 patients from four clinical trials (1547 patients in the experimental arms and 1500 in the control arms). In the ITT population, all the experimental arms showed an advantage compared with the standard of care arm (including the individual patient data of PFS from all the control arms of the clinical trials) (Figure 6 and Figure S1). This advantage was statistically significant for KEYNOTE-361 (HR 0.47 CI 95% 0.40–0.56, *p* < 0.001), EV302 (HR 0.49 CI 95% 0.42–0.57, *p* < 0.001), and CheckMate-901 (HR 0.73 CI 95% 0.62–0.85, *p* < 0.001), while there was no statistically significant advantage for IMVIGOR130 (HR 0.95 CI 0.84–1.08, *p* = 0.47).

#### 5.3.3. Comparison with Avelumab Data

We compared the obtained individual patient data from the included clinical trials to those obtained from the JAVELIN BLADDER 101 trial for OS and PFS (Figures S6–S9). In the OS analysis, no trials showed a statistically significant advantage against avelumab (analysis started after the first chemotherapy phase) (median OS 21.9 m (19—not estimable (NE)). In our analysis, EV302 had an HR of 0.81 (CI 95% 0.62–1.0, *p* = 0.12) with a median OS that was NE (NE–21.8); CHECKMATE-901 showed an HR of 1.18 (CI 95% 0.93–1.5, *p* = 0.172) with a median OS of 21.4 m (17.3–28.5); IMVIGOR130 had an HR of 1.61 (CI 95% 1.29–2.0, *p* < 0.001) with a median OS of 15.6 m (12.1–18.9); and KEYNOTE-361 had an HR of 1.63 (CI 95% 1.31–2.0, *p* < 0.001) with a median OS of 15.1 m (12.2–17.4). In terms of PFS, two trials showed a significant advantage versus avelumab (median PFS of 3.8 m (3.7–5.7): EV302 (HR 0.49, CI 95% 0.40–0.60, *p* < 0.001) with a median PFS of 16.5 m (11.3–NE) and KEYNOTE-361 (HR 0.48, CI 95% 0.38–0.61, *p* < 0.001) with a median PFS of 12.3 m (10.1–13.8). The other included trials had a non-significant advantage: CHECKMATE-901 (HR 0.82, CI 95% 0.67–1.01, *p* = 0.069) had a median PFS of 5.5 m (4.1–7.5) and IMVIGOR130 (HR 0.97, CI 95% 0.81–1.17, *p* = 0.759) had a median PFS of 5.3 m (4.5–6.5).

## 6. Safety 

For the safety analysis, we included 3040 patients from four studies (EV302, CHECKMATE-901, KEYNOTE-361, IMVIGOR130) and compared incidences for any grade neutropenia, anemia, cutaneous rash, or pruritus (Figure S10).

Considering any grade neutropenia, 438 events occurred in 1551 patients for the experimental arms (28%), versus 585 in 1489 patients (39%) for the control arms (odds ratio (OR) 0.64 CI 95% 0.54–0.75). EV302’s experimental arm is the arm with less neutropenia events (40 in 442 patients, 9%). In the experimental arms, any grade anemia occurred in 903 of 1551 patients (58%), while in the control arms it occurred in 881 of 1489 patients (59%), with an OR of 0.93, CI 95% 0.80–1.08.

For any grade cutaneous rash, the experimental arms had 337 events in 1551 patients (22%), while the control arms had 80 events in 1489 patients (5%), with an OR of 4.81, CI 95% 3.73–6.19. The EV–Pembrolizumab arm is the one with more cutaneous rash events (145 in 442 patients, 32%).

In terms of events of any grade pruritus, in the experimental arms there were 343 events in 1551 patients (22%) vs. 85 events in 1489 patients (5%) in the control arm, with an OR of 4.81 (CI 95% 3.74–6.18). The EV–Pembrolizumab arm was the one with more pruritus events, at a rate of 146 in 442 patients (33%).

## 7. Discussion

We have conducted the present meta-analysis in patients (unselected for PDL1 expression, or fits for cisplatin, unselected for PS ECOG status) who underwent first-line treatment for urothelial cell carcinoma with the objectives of (I) comparing first-line Pembrolizumab + EV versus other immunotherapy-based treatments; (II) confirming the efficacy and safety of immunotherapy-based combinations versus chemotherapy alone (platinum + Gemcitabine, defined as SoC); and (III) exploring the outcomes of the included trials compared to avelumab in the phase 3 JAVELIN BLADDER 101 trial, excluding from the analysis patients who progressed during the first 4 months of therapy in the included trials to select patients with a prognosis similar to the ones included in the JAVELIN BLADDER 101 trial [8].

Comparing the IPD obtained from the Kaplan–Meier curve for the OS and PFS of EV302 vs. the other clinical trials (KEYNOTE-361, IMVIGOR130, and CHECKMATE-901), Pembrolizumab + EV showed a statistically significant superiority in terms of OS and PFS, except for PFS in KEYNOTE-361. However, the KEYNOTE-361 trial does not meet its primary endpoint of superior PFS of Pembrolizumab plus chemotherapy vs. chemotherapy, and when comparing its standard of care arm with the other trials, it showed better results in terms of PFS (HR 0.47 CI 0.37–0.59 vs. CM-901 SOC arm, Figures S3 and S4), suggesting different prognoses or patient selections in the standard of care arm of KEYNOTE-361. Since the EV–Pembrolizumab combination is cisplatin free, it appeared particularly promising in all patients but even more in the 40/60% of patients who were considered unfit for cisplatin. Nevertheless, a subgroup analysis of EV302 [17] showed how there is no difference in terms of efficacy for cisplatin-eligible vs. -ineligible patients treated with Enfortumab vedotin–Pembrolizumab. According to the results of EV302, Pembrolizumab + EV was approved in the first-line setting by the FDA and is considered as the upcoming standard of care in the first-line setting.

Immunotherapy combinations in the first-line treatment of urothelial cell carcinoma were superior to chemotherapy alone in this analysis (even if we obtained a non-significant advantage for KEYNOTE-361 and IMVIGOR130 in terms of OS and non-significant advantage for IMVIGOR130 in terms of PFS). In particular, the combination of Pembrolizumab + EV reported a statistically significant advantage in terms of OS compared to SoC (HR 0.47, CI 0.38–0.58).

The current standard of care in patients who reported a response or stable disease after first-line platinum-based chemotherapy for four up to six cycles is represented by the addition of maintenance avelumab to best supportive care according to the JAVELIN BLADDER 101 trial [8]. With the addition of avelumab as a maintenance treatment in the first-line setting, patients obtained a reduction risk of 40% and 30% in progression-free and overall survival, respectively.

To evaluate if the new immunotherapy-based strategy was superior to the current SoC, we removed from the analysis patients who progressed in the first 4 months of the included clinical trials, we compared the IPD of these patients to the patients enrolled in the maintenance study, the JAVELIN BLADDER 101 trial.

The Pembrolizumab-based trials (EV 307 and KEYNOTE-361) showed a significantly better PFS than avelumab alone. However, this benefit is not reflected in a statistically significant advantage in terms of OS. Moreover, the other combination strategies (CHECKMATE-901, IMVIGOR130, KEYNOTE-361) seem to not be as good as avelumab maintenance (CHECKMATE-901 showed an HR of 1.18 (CI 95% 0.93–1.5, *p* = 0.172), IMVIGOR130 had an HR of 1.61 (CI 95% 1.29–2.0, *p* < 0.001), and KEYNOTE-361 had an HR of 1.63 (CI 95% 1.31–2.0, *p* < 0.001)).

From this analysis, an immuno-based combination strategy at the beginning of treatment seems to be superior in terms of PFS, but it is substantially similar to the current strategy in terms of OS, in which immunotherapy with avelumab is administered sequentially, after response/stability to 4/6 cycles of platinum-based chemotherapy. Although it is not statistically significant, we highlight that EV302 reports an absolute risk reduction in mortality by 19% compared to patients who followed avelumab maintenance (HR 0.81, CI 0.61–1.1). However, to confirm its superiority, a longer follow up is needed. Our analysis is exploratory, and the attempt to remove progressed patients from the IPD curves of the other trials to make their prognosis like the JAVELIN BLADDER 100 ones needs to be confirmed by prospective data as it is not possible to express a definitive evaluation; another interesting consideration is that the JAVELIN BLADDER 100 trial included only a few patients (n = 6) receiving EV after discontinuing avelumab maintenance therapy. Recently, the AVENANCE study [18], conducted in France, analyzed the subsequent treatments received by patients with avelumab maintenance therapy: the median OS from first-line platinum-based chemotherapy in patients with avelumab followed by ADC (antibody–drug conjugate) therapy (Enfortumab vedotin or Sacituzumab govitecan) was 41 months versus 24.5 months in patients who received second-line platinum-based treatment. These data support the effectiveness of ADCs, in particular Enfortumab vedotin, in urothelial cancer.

Considering safety analysis, the first-line chemo-immunotherapy combinations showed more G3–G4 adverse events compared to chemotherapy and these AE differences are mainly due to immune-related toxicities. Concerning the combination strategy of EV plus Pembrolizumab, we need to underline that there are different adverse events, especially peripheral neuropathy and cutaneous rash, that, even if manageable in most cases, could become severe and dose limiting. Cisplatin induces peripheral neuropathy too, but it tends to be reversible when stopping chemotherapy (4–6 cycles), differently from EV which is scheduled continuously, until progression/unacceptable toxicities.

The main limitations of our meta-analysis are the following: (i) We used reconstructed IPD, so we were unable to adjust patient-level covariates. Heterogeneity among the population of the trials may affect the pooled results (e.g., in CHECKMATE-901, only cisplatin-eligible patients were enrolled, differently from the other trials included; the standard of care arms of KEYNOTE-361 overperformed compared to the other arms). (ii) Data on peripheral neuropathy were reported only in the EV + Pembrolizumab study. (iii) The exploratory analysis comparing immunotherapy from the beginning versus maintenance is spoiled by a different selection of patients among the trials. Indeed, we selected patients from IPD curves after a time of four months from the start (approximately the time of four cycles of chemotherapy), while for the avelumab maintenance, patients were selected when no disease progression occurred (i.e., an ongoing complete response, partial response, or stable disease) after the receipt of four to six cycles of chemotherapy with Gemcitabine plus cisplatin or carboplatin; a treatment-free interval of 4 to 10 weeks since the last dose of chemotherapy was permitted.

According to our results, we suppose that Enfortumab vedotin plus Pembrolizumab will be the new standard of care in first-line settings for metastatic urothelial cancer in all comers, followed by a platinum-based chemotherapy regimen. However, further analyses are needed to confirm this hypothesis.

## 8. Conclusions

We reported an IPD meta-analysis that showed an OS superiority of EV + Pembrolizumab compared to the other immunochemotherapy combinations included in the present study. Safety data, as expected, showed worse myelosuppression in the immunochemotherapy combination and worse rash and pruritus in the EV–Pembrolizumab combination. Improved outcomes have been shown by using immunochemotherapy combinations rather than chemotherapy alone in the first-line setting of mUC (non-significant advantage for KEYNOTE-361 and IMVIGOR130 in terms of OS and non-significant advantage for IMVIGOR130 in terms of PFS). Compared to avelumab maintenance, EV–Pembrolizumab was superior in terms of OS. Further analyses and a longer follow up are recommended to confirm these results. 

### Clinical Practice Points

The first line of metastatic urothelial cancer (mUC) has been limited to chemotherapy for years, until the advent of avelumab maintenance in responsive patients with the JAVELIN BLADDER 101 trial and subsequent trials on immunotherapy combinations.

This IPD meta-analysis tries to compare first-line options for mUC, evidencing the impact of Enfortumab vedotin plus Pembrolizumab compared to other ICI combination strategies.

## Figures and Tables

**Table 1:** Main characteristics of included studies.

Study (Year)	Study Name (NCT Number)	Eligible Patients	Study Drug	No. of Patients	Cisplatin-Eligible Patients	Endpoints
Michiel S. van der Heijden et al., 2023 [9]	CheckMate-901 NCT03036098	first-line metastatic urothelial carcinoma	Nivolumab + Gemcitabine–Cisplatin vs. Gemcitabine–Cisplatin	304 vs. 304	608 (100%)	OS: 21.7 vs. 18.9 mo HR 0.78 [0.63–0.96] PFS: 7.9 vs. 7.6 mo OS (PDL1 ≥ 1%): HR 0.75 [0.53–1.06] PFS (PDL1 ≥ 1%): HR 0.60 [0.41–0.81] EORTC QLQ-C30: 40% vs. 66%
Galsky et al. 2018 [7]	IMVIGOR130 NCT02807636	first-line metastatic urothelial carcinoma	Atezolizumab + Gemcitabine–Cisplatin/Carboplatin vs. Atezolizumab vs. Placebo + Gemcitabine–Cisplatin/Carboplatin	451 vs. 362 vs. 400	261 (58%) vs. 191 (53%) vs. 224 (56%)	mPFS (A vs. C): 8.2 vs. 6.3 mo HR 0.72 [0.70–0.96] mOS (A vs. C): 16.0 vs. 13.4 mo HR 0.83 [0.69–1.00] OS (B vs. C): 15.7 vs. 13.1 mo HR 1.02 [0.83–1.24]
Powles et al. 2023 [16]	EV302 NCT-04223856	first-line metastatic urothelial carcinoma	Enfortumab Vedotin + Pembrolizumab vs. Gemcitabine–Cisplatin/carboplatin	442 vs. 444	240 vs. 242	PFS (EV + P vs. Chemo): 12.5 vs. 6.3 mo HR 0.45 [0.38–0.54] OS (EV + P vs. Chemo): 31.5 vs. 16.1; HR 0.47 [0.38–0.58] ORR (EV + P vs. Chemo): 67.7 vs. 44.4
Powles et al. 2018 [12]	KEYNOTE-361 NCT02853305	first-line metastatic urothelial carcinoma	Pembrolizumab + Gemcitabine–Cisplatin/Carboplatin vs. Pembrolizumab vs. Gemcitabine–Cisplatin/Carboplatin	351 vs. 307 vs. 352	156 (44%) vs. 137 (45%) vs. 156 (44%)	mPFS (pembro + chemo vs. chemo): 8.3 vs. 7.1 mo HR 0.78 [0.65–0.93] mOS (pembro + chemo vs. chemo): 17.0 vs. 14.3 mo HR 0.86 [0.71–1.02] mOS (pembro vs. chemo): 15.6 vs. 14.3 mo HR 0.92 [0.77–1.11] OS (CPS ≥ 10): 16.1 vs. 15.2 mo HR 1.01 [0.77 vs. 1.32]

## Data Availability

The data presented in this study are available on request from the corresponding author.

## Supplementary Materials

The following supporting information can be downloaded at: https://www.mdpi.com/article/10.3390/curroncol31080352/s1, Figure S1: Hazard ratios comparing progression free survival of the included trial comparing sperimental arm vs standard of care arm (which included individual patient data from all the included trials); Figure S2: Kaplan-meier Analysis of standard of care arms of the included trials; Figure S3: Hazard Ratios of standard of care arms compared each other; Figure S4: Kaplan meier analysis of Progression free survival in standard of care arms; Figure S5: Hazard Ratios of standard of care arms compared each other; Figure S6: Kaplan Meier Overall survival of experimental arms of stable or responder patients after the first 4 months compared to JAVELIN BLADDER 101; Figure S7: Hazard Ratio of overall survival of experimental arms of stable or responder patients after the first 4 months compared to JAVELIN BLADDER 101; Figure S8: Kaplan Meier progression freel survival of experimental arms of stable or responder patients after the first 4 months compared to JAVELIN BLADDER 101; Figure S9: Hazard Ratio of progression free survival of experimental arms of stable or responder patients after the first 4 months compared to JAVELIN BLADDER 101; Figure S10: Safety analysis.
