# Supplementary material for: Immunotherapy-Based Combinations in First-Line Urothelial Cancer: A Systematic Review and Individual Patient Data (IPD) Meta-Analysis
Source: Curr Oncol. 2024 Aug 20;31(8):4713–27. doi: 10.3390/curroncol31080352 (PMC11352654; doi:10.3390/curroncol31080352)
Supplement: Supplementary file 1 [file curroncol-31-00352-s001.zip › curroncol-3086943-supplementary.pdf]

Supplementary Figures

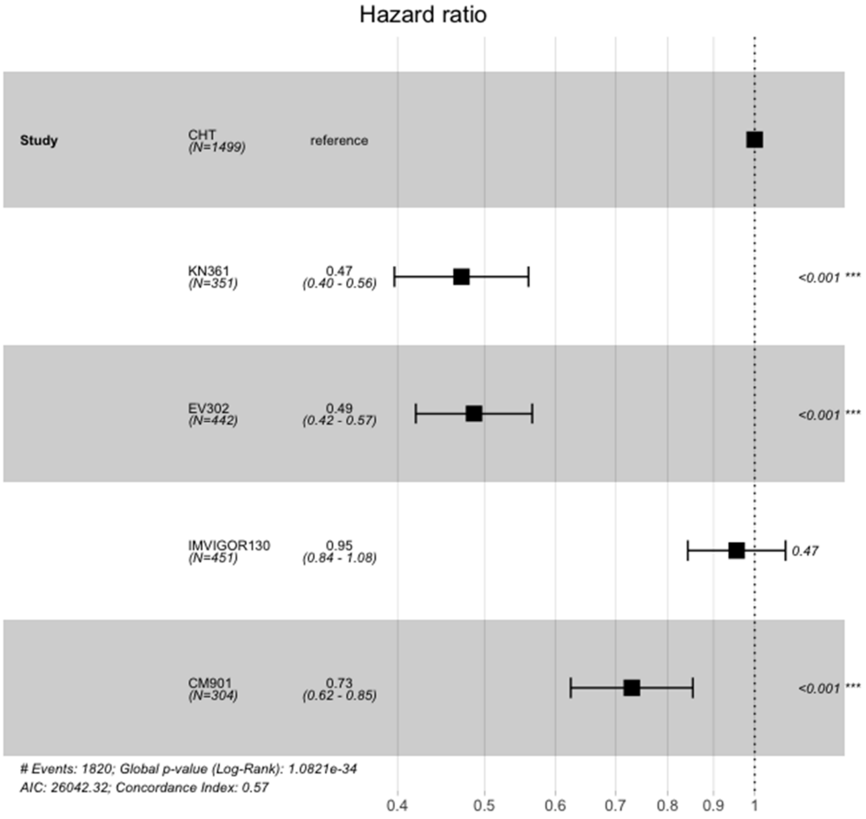

Figure S1. Hazard ratios comparing progression free survival of the included trial comparing sperimental arm vs standard of care arm (which included individual patient data from all the included trials).

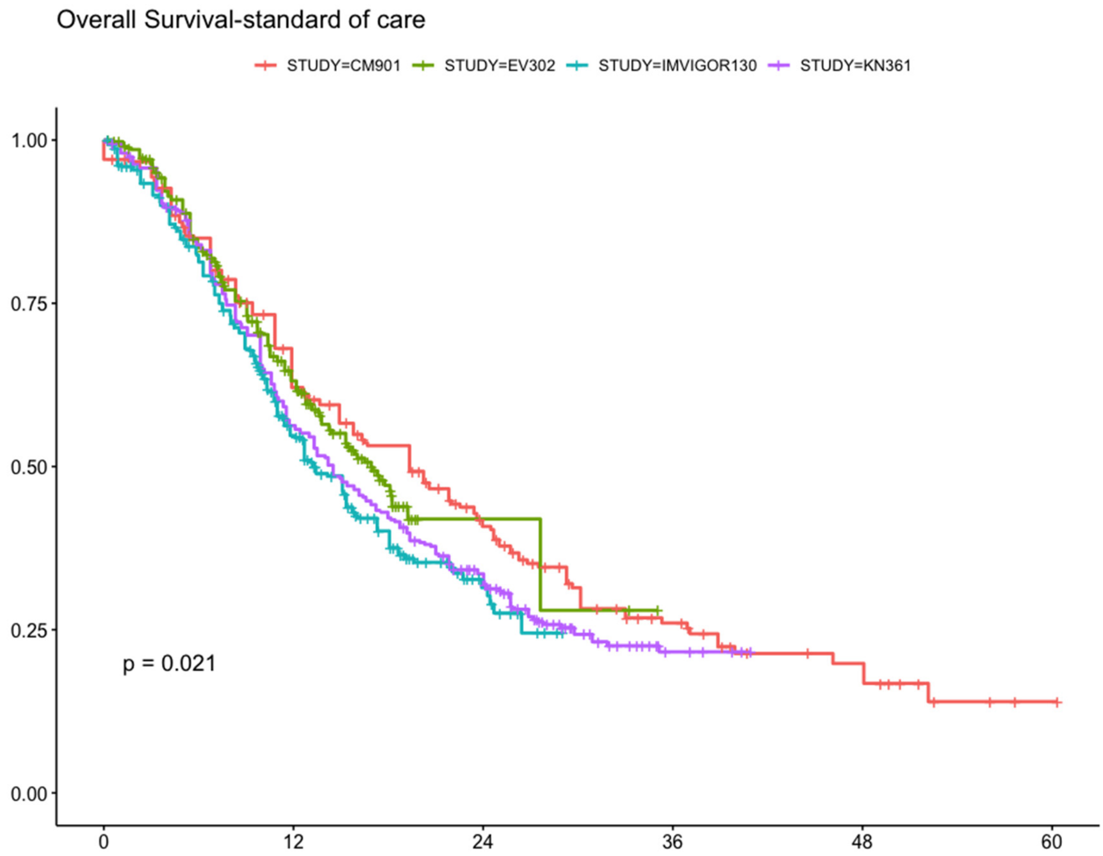

	0	12	24	36	48	60
STUDY=CM901	304	166	82	33	13	1
STUDY=EV302	444	164	3	0	0	0
STUDY=IMVIGOR130	400	182	25	0	0	0
STUDY=KN361	352	195	104	20	0	0

Figure S2. Kaplan-meier Analysis of standard of care arms of the included trials.

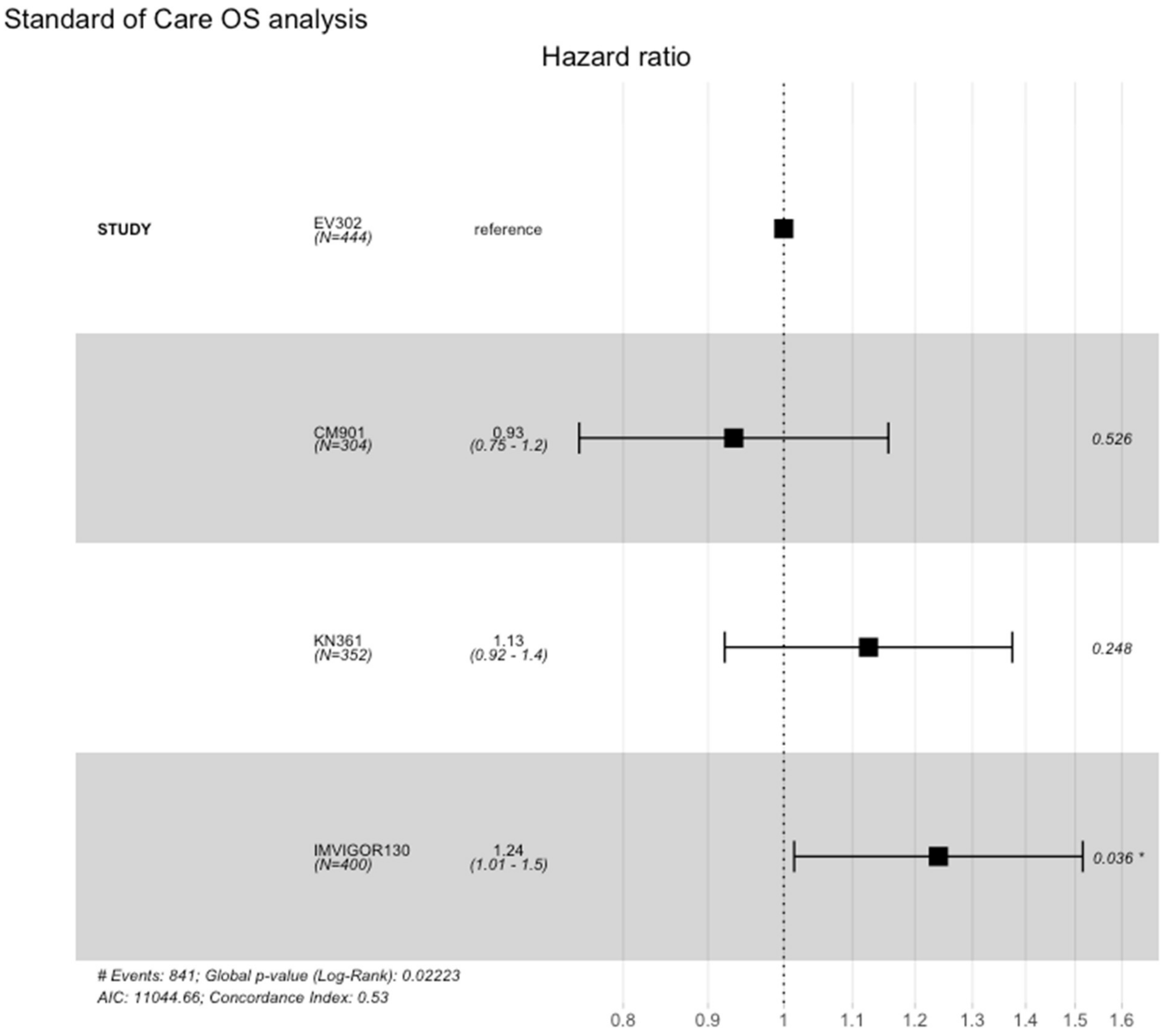

Figure S3. Hazard Ratios of standard of care arms compared each other.

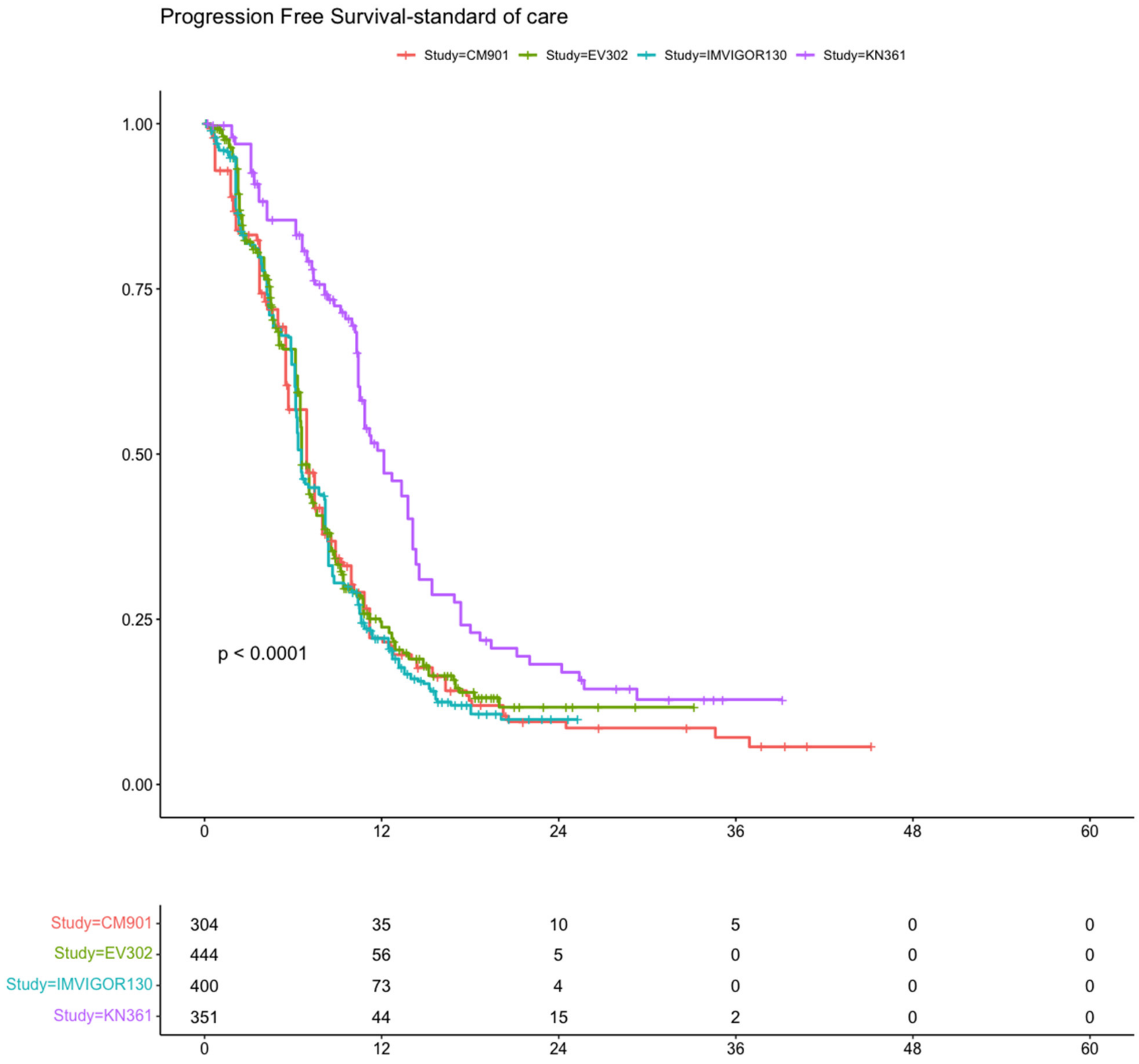

Figure S4. Kaplan meier analysis of Progression free survival in standard of care arms.

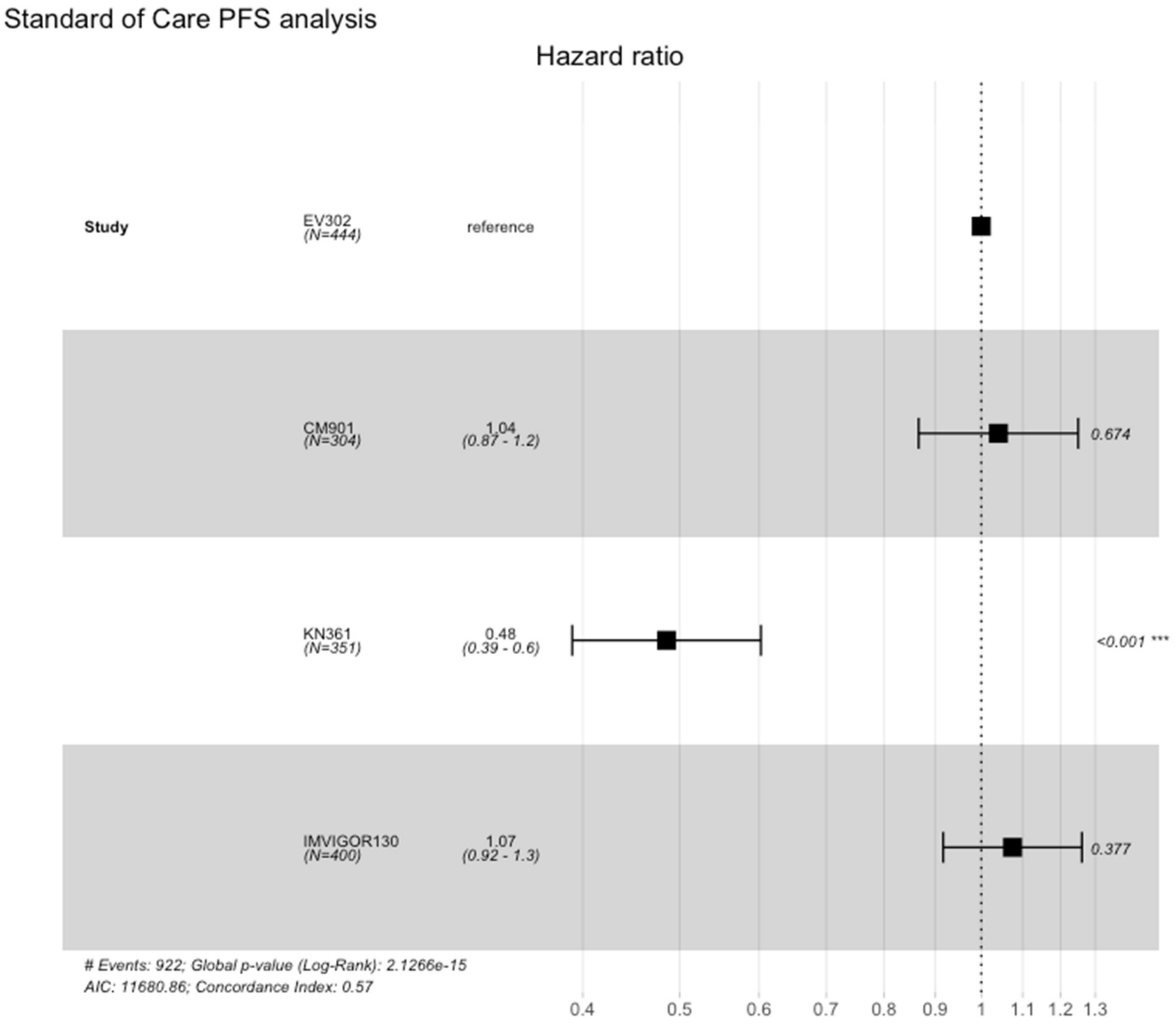

Figure S5. Hazard Ratios of standard of care arms compared each other.

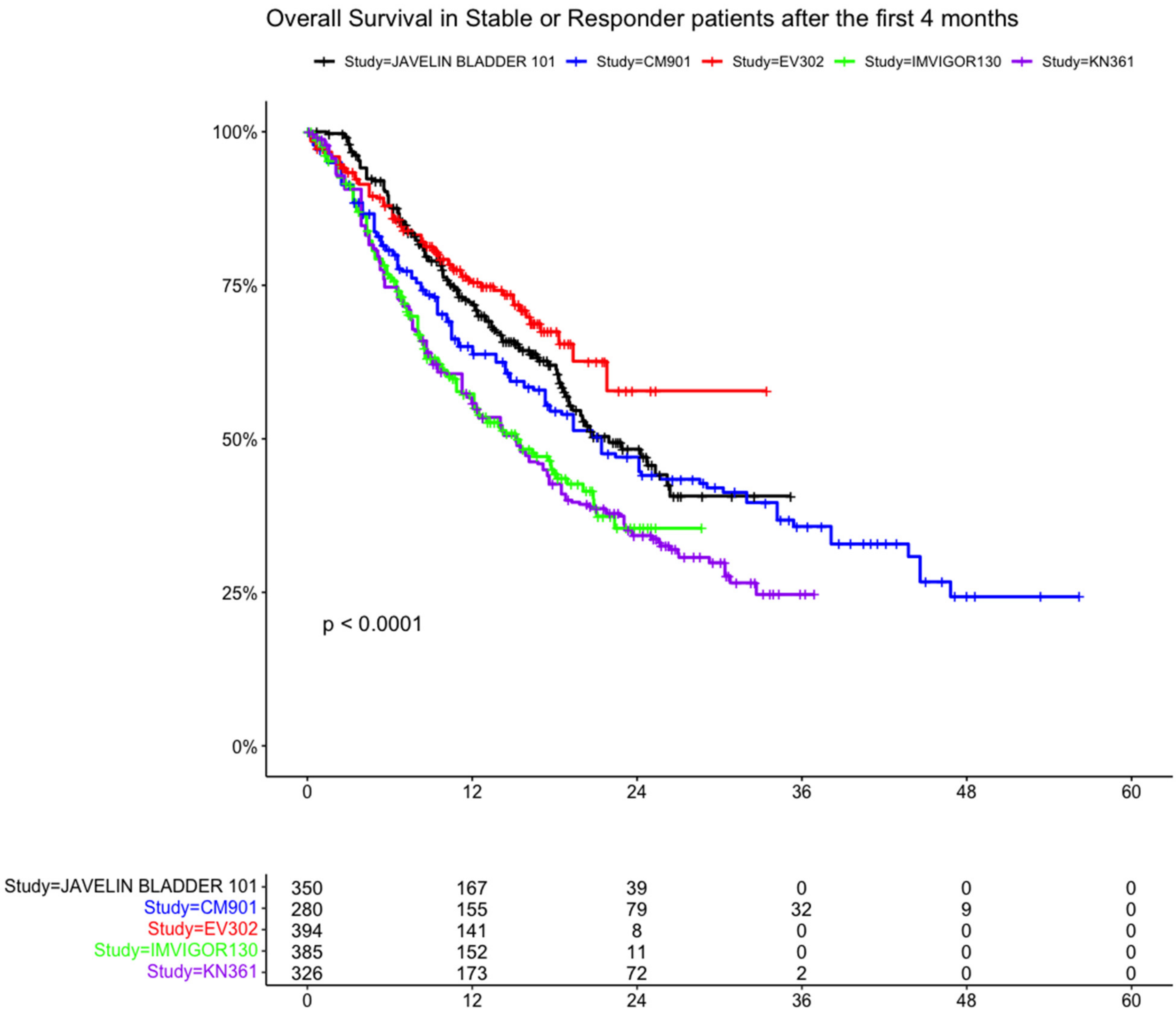

Figure S6. Kaplan Meier Overall survival of experimental arms of stable or responder patients after the first 4 months compared to JAVELIN BLADDER 101.

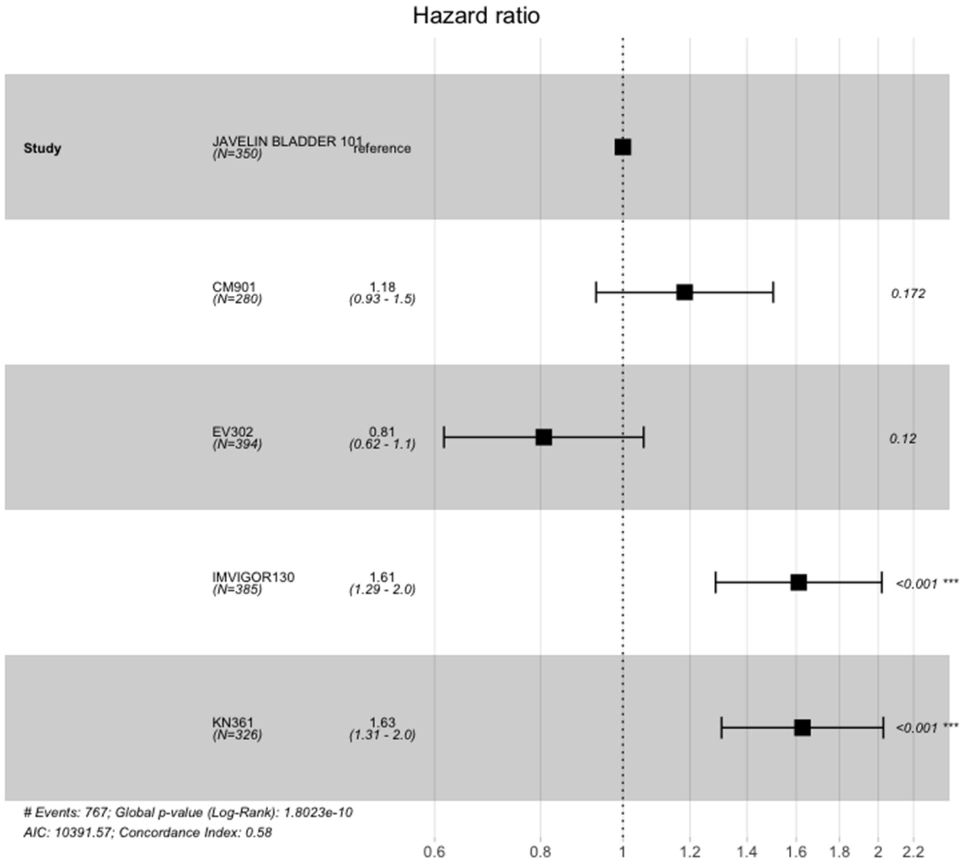

Figure S7. Hazard Ratio of overall survival of experimental arms of stable or responder patients after the first 4 months compared to JAVELIN BLADDER 101.

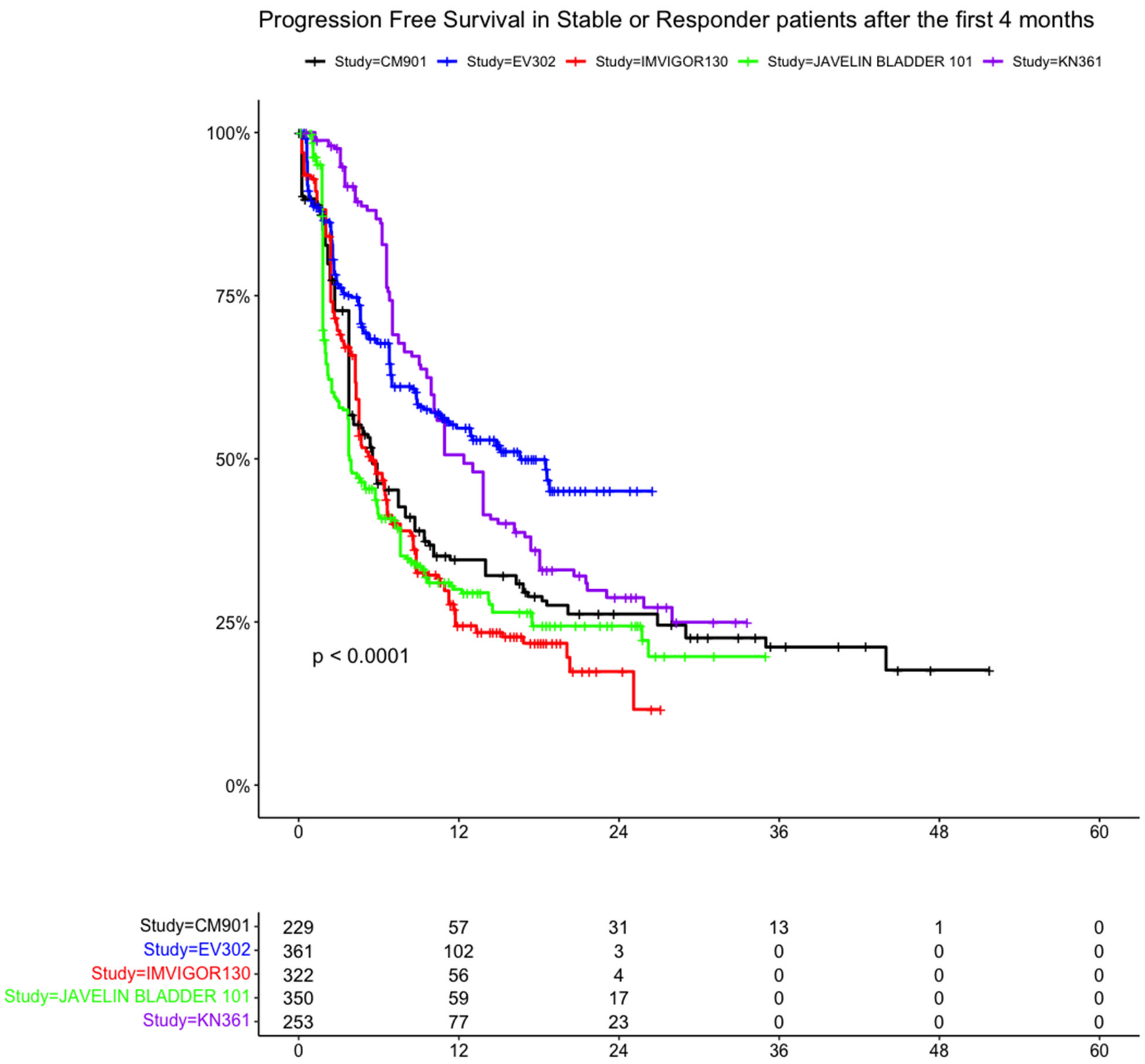

Figure S8. Kaplan Meier progression freel survival of experimental arms of stable or responder patients after the first 4 months compared to JAVELIN BLADDER 101.

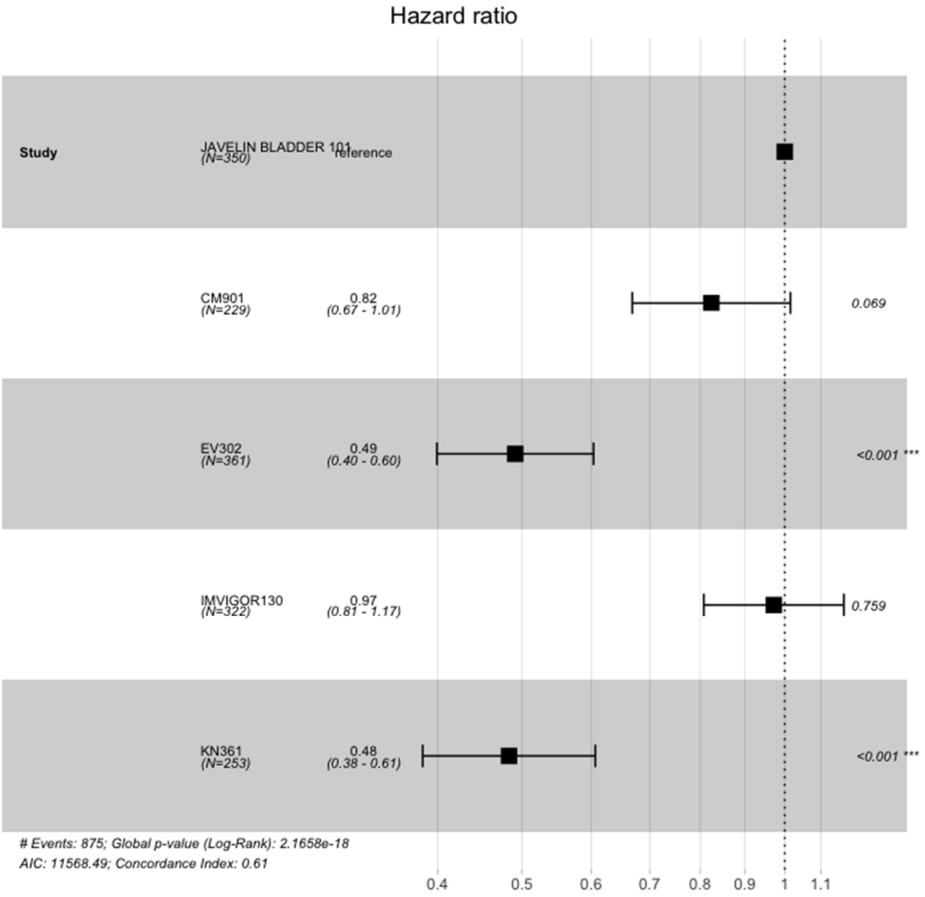

Figure S9. Hazard Ratio of progression free survival of experimental arms of stable or responder patients after the first 4 months compared to JAVELIN BLADDER 101.

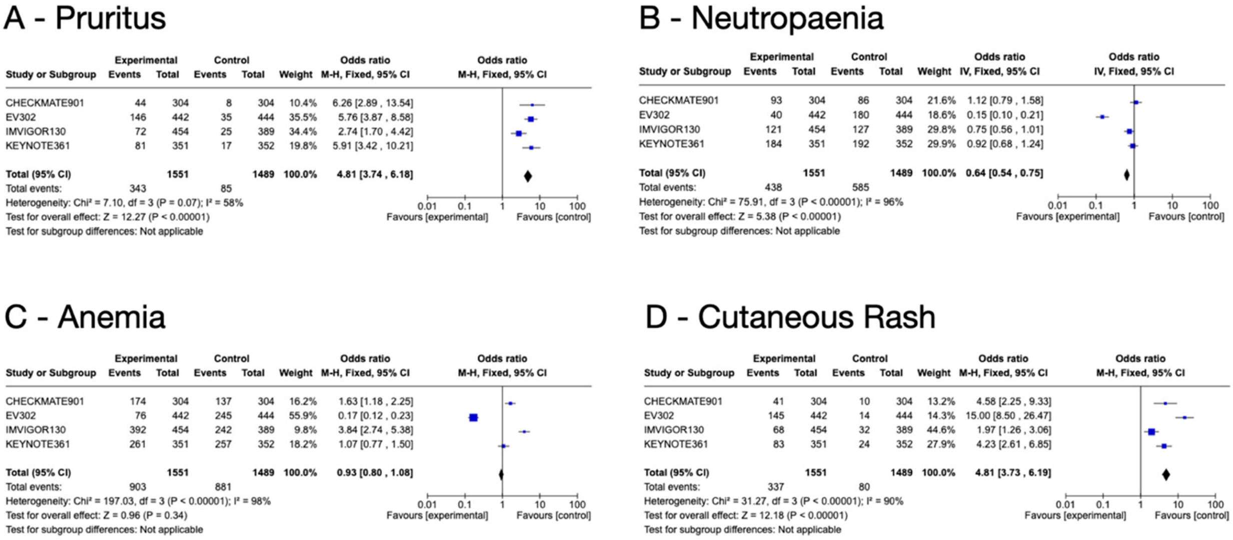

Figure S10. Safety analysis. A, Any grade pruritus in selected trials. B Any grade neutropenia in selected trials. C Any grade anemia in selected trials. D Any grade cutaneous Rash in selected trials.
